# Does Hemodynamic-Guided Heart Failure Management Reduce Hospitalization? A Systematic Review

**DOI:** 10.7759/cureus.1161

**Published:** 2017-04-13

**Authors:** Abdul M Minhas, Saba Ahmed, Muhammad S Khan, Kaneez Fatima, Muhammad N Anwar, Jonathan Constantin

**Affiliations:** 1 Internal Medicine, Orange Park Medical Center; 2 Medical Student, Dow University of Health Sciences (DUHS), Karachi, Pakistan; 3 Internal Medicine, John H Stroger J. Hospital of Cook County; 4 Civil Hospital, Dow University of Health Sciences (DUHS), Karachi, Pakistan; 5 Cardiology, Orange Park Medical Center

**Keywords:** heart failure, implantable hemodynamic sensors, hospitalization

## Abstract

Heart failure (HF) is a pressing health concern as the expense of hospitalization financially burdens the health care system. Hemodynamic monitoring has the potential to detect increases in intracardiac filling pressures weeks before clinical deterioration; hence, preliminary findings of volume overload with the use of these devices may prevent the progression of disease and lead to a reduction in HF-associated hospitalizations. We extensively searched PubMed, Ovid SP, Embase, and Cochrane databases to identify all the possible studies that assess the effect of hemodynamic monitoring on hospitalizations in HF patients. The main outcomes considered were the rate of HF hospitalization, mortality, quality of life, and improvement in New York Heart Association (NYHA) functional class in the monitored group. Seven studies met all the eligibility criteria and were incorporated in our systematic review. Out of the seven studies we reviewed, three studies inserted the sensor in the pulmonary artery, three in the right ventricle, and only one in the left atrium. On an average, the single study on the left atrium showed the highest reduction (59.0%) in HF hospitalization followed by the pulmonary artery (56.3%) and right ventricle (31.0%), respectively. Our systematic review demonstrates that the use of hemodynamic sensors in HF patients helps to reduce HF-related hospitalizations. Therefore, a combination of outpatient monitoring via the use of hemodynamic sensors and fluid management is needed to reduce HF hospitalizations and improve outcomes in HF patients.

## Introduction and background

Heart failure (HF) is the most frequent cause of hospitalization in people over the age of 65 with over one million admissions per year [1]. HF is a pressing health concern as the expense of hospitalization financially burdens the health care system. Around 70% of all direct and indirect costs created by HF patients are due to hospitalization and approximately $39.2 billion was spent on care for patients with HF in the United States in 2010 [2-3]. The persistently high hospital readmission rates and prevalence for HF call for further improvements to current care approaches [4].

Several treatment strategies have been employed and tested in randomized clinical trials for early detection of the worsening of symptoms in HF patients. These strategies include telemonitoring, home weight monitoring, thoracic impedance, and remote monitoring [5-8]. Although these parameters have been shown to improve outcomes, they have failed to demonstrate an effect on re-hospitalizations in patients with HF [9-11]. Therefore, new management strategies are needed.

More than 90% of hospitalizations in decompensated HF are due to congestion, suggesting that monitoring for congestion is vital in the long-term management of HF [12]. Relying solely on symptoms and physical findings of volume overload has proven ineffective in avoiding HF-related hospitalizations, as these are usually late manifestations of decompensated HF when the filling pressures are already substantially high and hospital admission is unavoidable. On the other hand, hemodynamic monitoring has the potential to detect increases in intracardiac filling pressures weeks before clinical deterioration because of the close relationship between volume and pressure in association with impaired volume regulation.

Implantable devices to monitor the cardiopulmonary filling pressures have been developed which focus on the pathophysiology of underlying HF [10, 13-18]. Hence, preliminary findings of volume overload with the use of these devices may prevent the progression of disease leading to a reduction in HF-associated hospitalizations. This management strategy has been tested in a variety of clinical trials. The aim of this systematic review is to establish, using these completed trials, whether hemodynamic monitoring using implantable sensors prevents HF hospitalizations and readmissions without causing any obvious safety concerns.

## Review

An extensive literature search of PubMed, Embase, Medline, and Cochrane library were done using the keywords "hemodynamic monitoring" AND "hospitalization" AND "heart failure" OR "pulmonary artery" OR "right ventricle" OR "left atrium". The search was conducted from the inception of these databases until November 2016. Only articles written in English and published in peer-reviewed journals were included. Additional relevant articles were found by scrutiny of the bibliographies of the articles found in the search. Experts in the field were contacted to identify anything missed by the search.

All articles assessing the effect of hemodynamic monitoring on the hospitalizations in HF patients were considered. There was no restriction on the sample size, mean follow-up time, or type of study. Both types of studies, with and without a control group, were included. Eligible studies included patients with New York Heart Association (NYHA) Functional Class II, III and IV HF, regardless of the left ventricular ejection fraction. The main outcomes considered were the rate of HF-hospitalization, mortality, quality of life, and improvement in NYHA symptom class in the monitored group.

**Figure 1:**
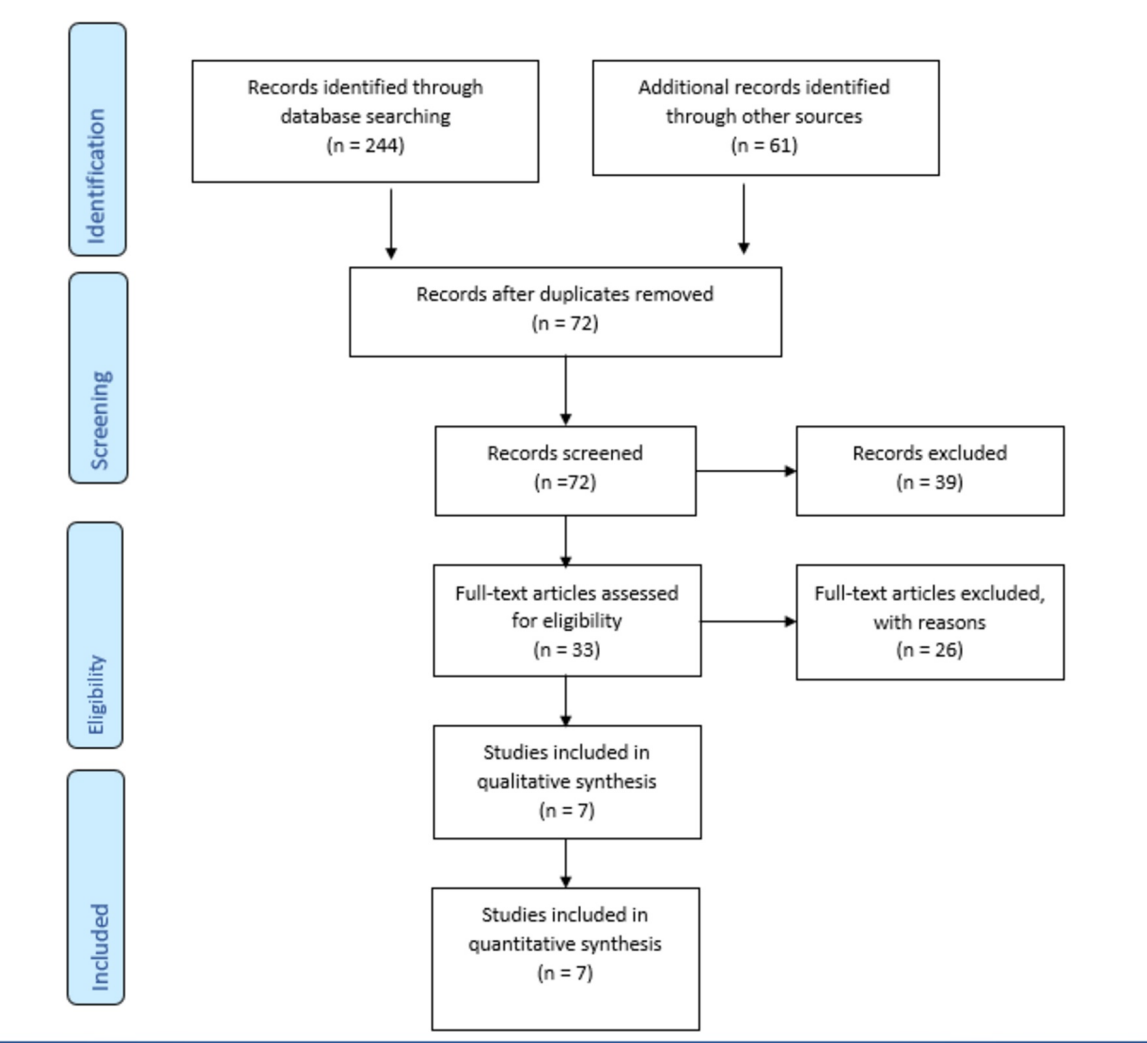
PRISMA Flow Sheet PRISMA: Preferred Reporting Items for Systematic Reviews and Meta-Analyses

Firstly, the titles and abstracts of all the retrieved articles were reviewed by two authors. Full texts of the articles that met the eligibility criteria were extracted. These articles were then independently reviewed by the two authors. Discrepancies regarding the eligibility of the studies were resolved by consensus. A third author was contacted in situations where these discrepancies could not be resolved. The detailed search strategy is outlined using the Preferred Reporting Items for Systematic Reviews and Meta-Analyses (PRISMA) flow sheet in Figure 1.

From the selected articles, descriptive information including the authors’ names, patient demographics, medical treatment, NYHA Class, outcomes, and methodology were collected. Any disagreement between the two authors over the risk of bias in the included studies was resolved by discussion or by consulting a third author. The details of the methodology involved in each trial were important for efficient comparison of the studies and interpretation of results. Handling of the competing outcomes of HF-hospitalization by the authors was assessed. The authors of the included trials were contacted whenever any data needed clarification or any additional information was required.

Seven studies met all the eligibility criteria and were incorporated in our systematic review. Baseline characteristics of the included articles are shown in Table 1. All the studies were conducted in the USA; however, one study was multicentric across Australia, New Zealand, and the USA [12]. All the included studies were prospective in nature and the majority (n=4) of them were randomized controlled trials (RCTs). Five out of seven studies had a control group. The number of participants ranged from 32 to 550 and were predominantly older, averaging around 60 years of age overall. Around two-thirds of the patient populations in all the included studies were male, except one [18]. All the studies included patients that had some residual symptoms of HF (NYHA Class II-IV).

**Table 1:** Characteristics of the Included Studies I: Intervention group; C: Control group; NYHA: New York Heart Association; US: United States; RCT: random controlled trials

Serial #	First author, year	Study design	Location	Total number of subjects (N)	I and C: N=	Age (I/C)	Percentage of males (I/C; %)	NYHA Class
1.	Bourge, 2008 10]	Prospective, single-blind, parallel, RCT trial	US	274	[I] N=134 [C] N=140	58±14/58±13	66/64	III-IV
2.	Ritzema, 2010 [12]	Prospective, observational, open-label.	US/ Australia, New Zealand	40	[I] N=40 [C] N=0	66±10	78/0	III
3.	Adamson, 2003 [18]	Prospective, observational, historic control.	US	32	[I] N=32 [C] N=0	59±10	38/0	II-III
4.	Jermyn, 2016 [19]	Prospective, case series with comparison to concomitant control.	US	66	[I] N=34 [C] N=32		76/59	III
5.	Adamson, 2011 [20]	Prospective, single-blind, RCT.	US	400	[I] N=202 [C] N=198	55±15/55±15	70/67	II-III
6.	Abraham, 2011 [21]	Prospective, single-blind, RCT.	US	550	[I] N=270 [C] N=280	61±13/62±13	72/73	III
7.	Abraham, 2016 [22]	Prospective, single-blind, RCT.	US	550	[I] N=270 [C] N=280	61.3±13/61·8	72/73	III

The primary outcome assessed in all the studies was the rate of HF-related hospitalizations (Table 2). The duration of mean follow-up ranged from six to 25 months overall. The devices used included an implantable left atrial pressure (LAP) monitoring system, implanted right ventricle (RV) intracardiac continuous hemodynamic monitor, and CardioMEMS™ Heart Sensor (St. Jude Medical, Inc, Atlanta, GA). The study by Jermyn, et al. [19] showed the highest reduction in hospitalizations (84%), whereas the study by Adamson, et al. [20] showed no reduction in the number of hospitalizations (0.004%). Two studies [12, 18] showed more than a 50% reduction while the remaining studies [10, 21-22] were less than 50%.

**Table 2:** Effectiveness of Hemodynamic Sensors in Reducing Hospital Readmissions in Patients with Chronic Heart Failure HF: heart failure; I: Intervention group; C: Control group; LAP: left atrial pressure; RV: right ventricle; ICD: implantable cardioverter-defibrillator

Serial #	First author, year	N= Intervention group [I] and Control-group [C]	Device used	Mean follow-up (months)	Reductions in HF hospitalizations (%)	p-value
1.	Ritzema, 2010 [12]	[I] N=40, [C] N=0	Implantable LAP monitoring system	25	59.0	0.041
2.	Abraham, 2011 [21]	[I] N=270, [C] N=280	CardioMEMS™​ Heart Sensor	15	37.0	
3.	Adamson, 2003 [18]	[I] N=32, [C] N=0	Implanted RV intracardiac continuous hemodynamic monitor	17	57.0	< 0.01
4.	Bourge, 2008 [10]	[I] N=134, [C] N=140	Implanted RV intracardiac continuous hemodynamic monitor	6	36.0	0.03
5.	Adamson, 2011 [20]	[I] N=202, [C] N=198	Implanted ICD with RV intracardiac continuous hemodynamic sensor	12	0.004	
6.	Jermyn, 2016 [19]	[I] N=34, [C] N=32	CardioMEMS™ Heart Sensor	15	84.0	
7.	Abraham, 2016 [22]	[I] N=270 [C] N=280	CardioMEMS™​ Heart Sensor	13	48.0	< 0.0001

Table 3 illustrates the differences in the rate of hospitalizations according to the location of the devices. Out of the seven studies we reviewed, three studies inserted the sensor in the pulmonary artery, three in the right ventricle, and only one in the left atrium. On an average, the single study on the left atrium showed the highest reduction (59.0%), followed by the pulmonary artery (56.3%) and right ventricle (31.0%), respectively. No major pressure sensor failures were reported by any of the included studies.

**Table 3:** Differences in Rates of Hospitalization According to the Location of the Device HF: heart failure

Location of device	First author, year	Reductions in HF hospitalizations (%)	Mean reduction (%)
Pulmonary artery	Abraham, 2011 [21]	37.0	56.3
	Jermyn, 2016 [19]	84.0	
	Abraham, 2016 [22]	48.0	
Right ventricle	Adamson, 2003 [18]	57.0	31.0
	Bourge, 2008 [10]	36.0	
	Adamson, 2011 [20]	0.004	
Left atrium	Ritzema, 2010 [12]	59.0	59.0

In this systematic review, we observed that the use of hemodynamic sensors in HF patients lowers the rate of HF hospitalizations when compared to usual care. These trials included devices that measure pulmonary artery pressure, right ventricular pressure, and left atrial pressure. The studies were in congruence that monitoring filling pressures aids in avoiding exacerbations of HF that lead to hospitalizations; two studies reported that the filling pressures in patients experiencing an HF event were significantly elevated regardless of treatment group (p < 0.001) [20] and that there was an increase in the pressure 24 hours before hospitalization [18]. These findings are in harmony with previously published research [23-26].

Jermyn, et al. reported the highest rate of reduction in HF-hospitalizations (84%) among all seven studies. This study used the patients in the monitored group as their own controls by comparing their rates of hospitalizations in the year prior to the implantation of the sensor [19]. Comparing the same patient population before and after implantation may be more effective as the severity of underlying HF or various co-morbidities vary in different sets of patients and may alter the results. The results of this study may also have been exaggerated due to a small sample size. On the contrary, Adamson, et al. reported no reduction in the rate of HF hospitalization in the 12 months of follow-up [20]. However, this study was terminated earlier than planned during early enrollment as a device failure was observed in the patients from previous trials that used the same pressure sensing lead. Early enrollment termination and the defective pressure sensing lead may have undermined the results of the trial.

Along with reducing the rate of HF events, Abraham, et al. found a significant decrease in the length of HF hospitalizations (p = 0.02) with the use of hemodynamic monitoring [21]. Furthermore, Abraham, et al. and Ritzema, et al. found an improvement in the quality of life and mortality in patients receiving hemodynamic care [12, 21]. On the other hand, another study reported no significant reduction in the rate of mortality in these patients (p = 0.23) [22]. NYHA symptom class improved significantly in the study by Jermyn, et al. (p < 0.0001) [21]. Further assessment on the effect of hemodynamic sensors on the quality of life and mortality is imperative and recommended.

Bourge, et al. observed a lower than expected event rate in the control group [10]. This was attributed to the fact that regular patient contact with HF management teams reduces hospitalizations as suggested by previous research [27-28]. Therefore, a combination of hemodynamic monitoring via sensor implantation with frequent patient contact with the HF management team may optimize the outcomes in HF-related hospitalizations. Furthermore, Bourge, et al. reported that there was no increase in HF events because of over-diuresis; one of the major feared complications of using hemodynamic sensors. Ritzema, et al., who observed that awareness of pressure trends leads to a better compliance with prescriptions and appropriate diuretic dosing [12], supported this. Due to the association of high diuretic intake and poor HF outcomes, this may contribute to better outcomes and a decrease in the rate of HF hospitalization.

Several limitations in this review need to be considered. Our search was limited to articles in English and electronic databases which may have led to a language and publication bias and some pertinent articles not being included in the review. Our study may be affected by a population bias as all the trials were conducted in Western countries. The mean follow-up times for all the studies were different; the long-term effectiveness of these sensors may not have been assessed properly in those with a shorter follow-up time. The trials made use of different devices, and the individual efficacy of the devices may have affected the results of individual trials.

## Conclusions

The use of hemodynamic sensors in HF patients helps to reduce HF-related hospitalizations. Hemodynamic information is vital because patients, in whom filling pressures are increasing, are at a high risk of hospitalization. Therefore, a combination of outpatient monitoring via the use of hemodynamic sensors and fluid management is needed to reduce HF hospitalizations and improve outcomes in HF patients.
